# Tc-99m GSA scintigraphy within the first 3 days after admission as an early predictor of outcome in severe acute liver injury

**DOI:** 10.1038/s41598-021-92058-6

**Published:** 2021-06-15

**Authors:** Yuji Suzuki, Keisuke Kakisaka, Takuro Sato, Ryouichi Mikami, Hiroaki Abe, Tokio Sasaki, Yasuhiro Takikawa

**Affiliations:** Division of Hepatology, Department of Internal Medicine, Iwate Medical University School of Medicine, 1-1-1 Idaidori, Yahaba-cho, Shiwa-gun, Iwate 028-3694 Japan

**Keywords:** Liver diseases, Gastroenterology, Hepatitis

## Abstract

Patients with severe acute liver injury (SLI) usually recover spontaneously. However, some SLI patients progress to acute liver failure with varying degrees of hepatic encephalopathy. Acute liver failure is associated with high mortality and can be substantially reduced by liver transplantation. Therefore, distinguishing SLI patients who might progress to acute liver failure and are at a risk of death is important when evaluating patients needing liver transplantation. The present study aimed to determine whether technetium-99m-diethylenetriaminepentaacetic acid galactosyl human serum albumin (Tc-99m GSA) scintigraphy can predict the prognosis of patients with SLI. This prospective observational study included 69 SLI patients. The accuracy of Tc-99m GSA for predicting death or liver transplantation for 6 months was assessed. Between the two groups of patients stratified based on the cut-off values from the receiver operating characteristic curves, 6-month transplant-free survival was compared. Sixteen (23.2%) patients died or underwent liver transplantation from admission (poor outcome). The hepatic accumulation index was calculated by dividing the radioactivity of the liver region of interest by that of the liver-plus-heart region of interest at 15 min (i.e., LHL15). The LHL15 in the 16 patients (0.686) was significantly lower than that in survivors (0.836; *P* < 0.0001). The optimal LHL15 cut-off for distinguishing poor outcome and survival was 0.737 with a sensitivity of 81.3%, specificity of 88.7%, and area under the curve of 0.907 (95% CI, 0.832–0.981). When patients were divided into two groups based on the LHL15 cut-off value, the 6-month transplant-free survival was significantly lower in patients with an LHL15 level ≤ 0.737. Tc-99m GSA scintigraphy may help predict the prognosis of patients with SLI.

## Introduction

Patients with severe acute liver injury (SLI) without pre-existing chronic liver disease usually recover spontaneously. However, some SLI patients progress to acute liver failure (ALF), which is characterized by coagulopathy and hepatic encephalopathy (HE) due to an abrupt loss of hepatic function^1^. The survival rate of ALF has improved with medical management over the last three decades, but mortality remains high^2^. Emergency liver transplantation is often the only life-saving treatment available for patients with ALF resistant to medical treatment^3^. Numerous studies have proposed prognostic models for ALF using clinical and laboratory parameters to determine the likelihood of death or survival without liver transplantation^4–9^. However, only a few studies have attempted to develop methods for determining the outcomes of patients with SLI^10,11^. Recently, some observational studies have evaluated the natural history of SLI patients who do not fulfill the criteria for ALF upon initial examination. Some SLI patients progress to ALF with the development of HE, followed by death or need for liver transplantation^1,12,13^. Therefore, determining which SLI patients are at risk for progression to ALF and have poor prognosis is important for the early evaluation of patients requiring liver transplantation.

Asialoglycoprotein binds to asialoglycoprotein receptors, which exist exclusively on hepatic cell membranes, followed by absorption through receptor-mediated endocytosis and delivery to lysosomes for degradation^14^. Technetium-99m-diethylenetriaminepentaacetic acid galactosyl human serum albumin (Tc-99m GSA) is a synthetic asialoglycoprotein used to visualize and quantify hepatic binding^15^. Thus, hepatic receptor imaging using Tc-99m GSA enables quantification of hepatic function through receptor and blood clearance indices. The parameters obtained from Tc-99m GSA scintigraphy are objective tools for the assessment of liver function in patients with ALF, cirrhosis, and/or liver surgery^16–18^. Tc-99m GSA scintigraphy data are also preferable because they provide a reliable evaluation of hepatic function without being affected by the use of fresh frozen plasma for the treatment of coagulopathy.

To date, only one study has reported the usefulness of Tc-99m GSA scintigraphy for prognosis evaluation in ALF patients^16^, and none have evaluated its use for prognosis evaluation in SLI patients. To address this issue, we prospectively collected Tc-99m GSA scintigraphy data from patients with SLI to determine its use in the early identification of SLI patients at risk for poor prognosis.

## Methods

### Participants

**Figure 1:**
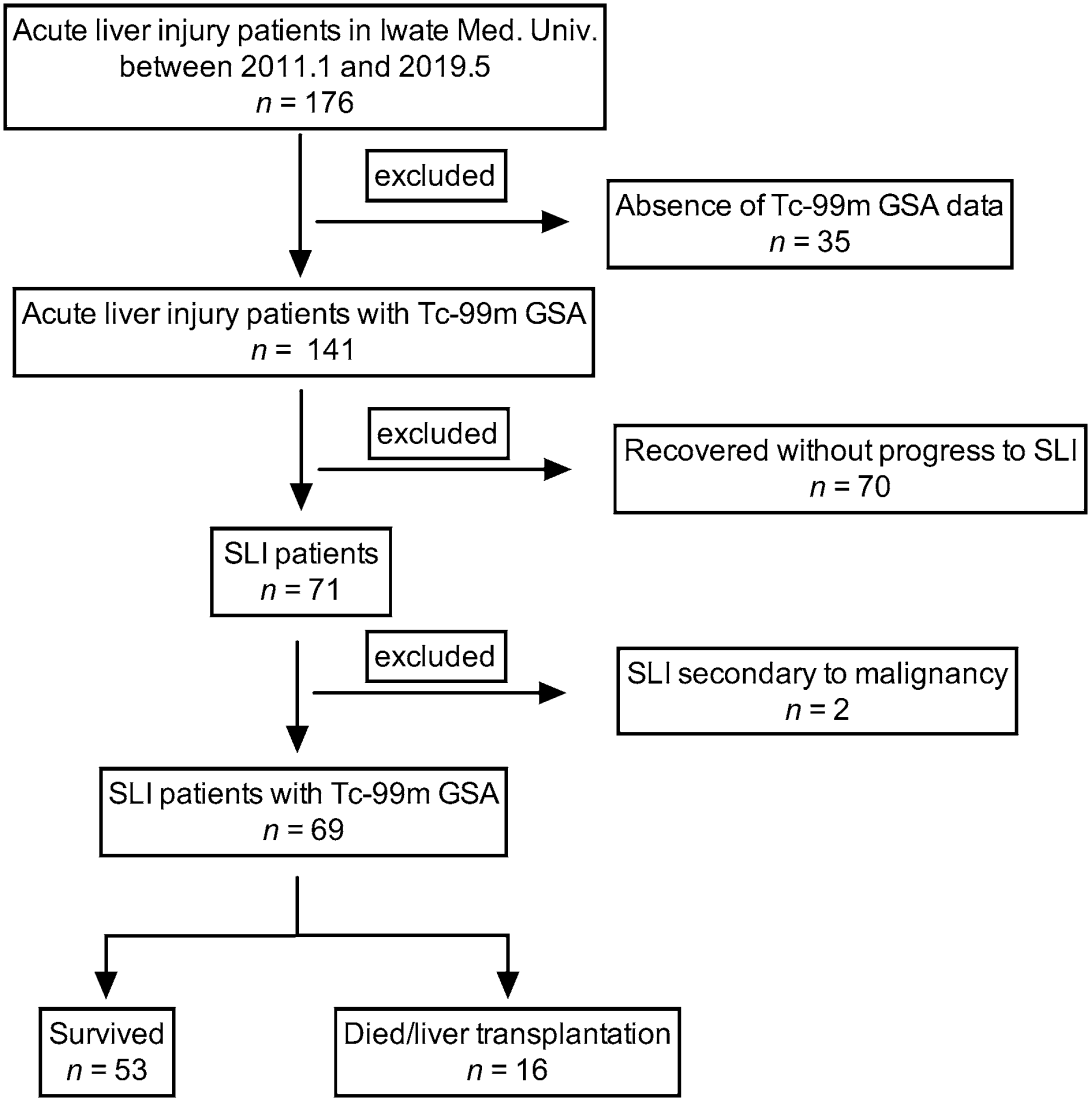
Flowchart of patient enrollment to creation of the study group.

This study included 176 patients with acute liver injury who were registered consecutively at our institution between January 2011 and May 2019. Acute liver injury patients with an aspartate aminotransferase (AST) level of > 200 IU/L or an alanine aminotransferase (ALT) level of > 300 IU/L in the absence of a pre-existing chronic liver disease were included in the study^19^. SLI patients were defined as those with acute liver injury who have a prolonged prothrombin time-international normalized ratio (PT-INR) over 1.5 without HE at the time of admission. Among the registered patients, 71 satisfied the inclusion criteria for SLI (Fig. 1). Two patients were excluded because of the presence of SLI secondary to malignant infiltration of the liver. Therefore, 69 patients were enrolled in the present study. Of note, SLI caused by acetaminophen overdose was not reported during the observational period. All patients were hospitalized because of symptoms of SLI. Data on patient characteristics (age, sex, date of hospital admission, date of first symptoms, and onset of jaundice and encephalopathy), etiology of the acute illness, need for mechanical ventilation, and renal replacement therapy were collected. The diagnosis of HE was based on the West Haven criteria^20^. The etiology was assessed based on historical, clinical, laboratory, and radiographic data. Liver biopsy is not mandatory for assessment of the etiology. Thirty-two of 69 patients underwent percutaneous liver biopsy after improvement of coagulopathy, to differentiate between autoimmune hepatitis and drug-induced liver injury or exclude chronic liver disease. The etiology was determined by discussion among at least three expert hepatologists.” All protocols reported were approved by the institutional review board of our institution (approval no.: H20-36). Informed consent was obtained from all participants. The present study was designed and conducted in accordance with the relevant guidelines and regulations of the ethical principles for medical research involving human subjects, as stated by the WMA Declaration of Helsinki.

### Procedures for Tc-99m GSA scintigraphy

Tc-99m GSA scintigraphy was performed by injecting 3 mg of Tc-99m GSA [185 MBq (5 mCi); Nihon Medi-Physics, Nishinomiya, Japan] into the cephalic vein. Images were obtained over 20 min with 15-s frames using a dual-head gamma camera (E.CAM; Toshiba, Tokyo, Japan) that was attached to a low-energy, high-resolution parallel-hole collimator. Time-activity curves were generated from the heart and liver regions of interest (ROIs). The hepatic accumulation index was calculated by dividing the radioactivity of the liver ROI by that of the liver-plus-heart ROI at 15 min (i.e., LHL15)^21^. The blood clearance index was calculated by dividing the radioactivity of the heart ROI at 15 min by that at 3 min (HH15).

### Statistical analysis

The laboratory data of 69 patients, including measures of liver injury and dysfunction (serum total bilirubin, aminotransferases, and PT-INR), were collected at the time of admission. The Model for End-Stage Liver Disease (MELD) scores were calculated using the patient’s creatinine (Cre), total bilirubin (T-Bil), and PT-INR levels at the time of admission based on the following formula: MELD = 9.57 log_e_ [Cre (mg/dL)] + 3.78 log_e_ [T-Bil (mg/dL)] + 11.20 log_e_ [PT-INR] + 6.43. The LHL15 and HH15 values calculated by Tc-99m GSA scintigraphy performed within 3 days after admission were used in the analysis.

Descriptive statistics were expressed as medians and interquartile ranges, and *n* (%) for continuous and categorical variables. For bivariate analysis, Student’s unpaired, two-tailed *t*-test or the Wilcoxon rank-sum test was used to compare continuous variables between patients who died or underwent liver transplantation and those who survived. Categorical variables were compared using the chi-squared test between patients who died or had liver transplantation and those who survived. One-way analysis of variance (ANOVA) using Bonferroni adjustments was used to identify the differences in LHL15 values between the groups. Transplant-free survival curves up to 6 months were estimated using the Kaplan–Meier method and compared using the log-rank test. Differences were considered significant when the *P*-value was < 0.05. Statistical analyses were performed using the JMP11 software package (SAS Institute, Cary, NC, USA). Graphs and figures were created using GraphPad Prism version 6 (GraphPad Software, San Diego, CA, USA). Although statistical analysis methods partially overlapped in our previous study^22^, the disease and analysis populations were different.

## Results

### Clinical characteristics

The baseline characteristics of the patients are shown in Table 1. Of the 69 patients, 31 were men and 38 were women, with a median age at diagnosis of 57.0 years. Among the 16 patients who died or underwent liver transplantation, 6 received transplantation. Liver transplantation was performed assuming that the patient would have died without it. The mean time from admission to Tc-99m GSA scintigraphy in this study was 2.4 (standard deviation, 0.93) days. The median (interquartile range) values for LHL15 and HH15 were 0.826 (0.725–0.876) and 0.768 (0.705–0.817), respectively. Typical Tc-99m GSA scintigraphy images of a patient without SLI with a normal LHL15 value and those of SLI patients are shown in Supplementary Fig. S1.

**Table 1:** Patient demographics.

	n = 69
Age, median (y)	57.0 ± 16.5 (39.5–68)
Male (%)	31 (44.9%)
**Etiology**	
Autoimmune	14
Drug	7
HBV	11
Other virus	8
Unknown	21
Other	8
Died or underwent liver transplantation, n (%)	16 (23.2%)
Survived without liver transplantation, n (%)	53 (76.8%)
Received liver transplantation, n (%)	6 (8.7%)
AST (U/L)	810 (234–2016)
ALT (U/L)	1076 (396–2133)
Total bilirubin (mg/dL)	10.7 (3.38–18.0)
PT-INR	1.65 (1.45–2.16)
MELD score	10.9 (8.9–12.6)
LHL15	0.826 (0.725–0.876)
HH15	0.768 (0.705–0.817)

Data are presented as *n* (%) or median (interquartile range [IQR]).

*HBV* hepatitis B virus, *AST* aspartate aminotransferase, *ALT* alanine aminotransferase, *PT-INR* prothrombin time-international normalized ratio, *MELD* model for end-stage liver disease, *LHL15* hepatic accumulation index calculated by dividing the radioactivity of the liver regions of interest by that of the liver-plus-heart regions of interest at 15 min; HH15, blood clearance index calculated by dividing the radioactivity of the heart regions of interest at 15 min by that at 3 min.

### Tc-99m GSA scintigraphy of patients who died or underwent liver transplantation and survived

**Figure 2:**
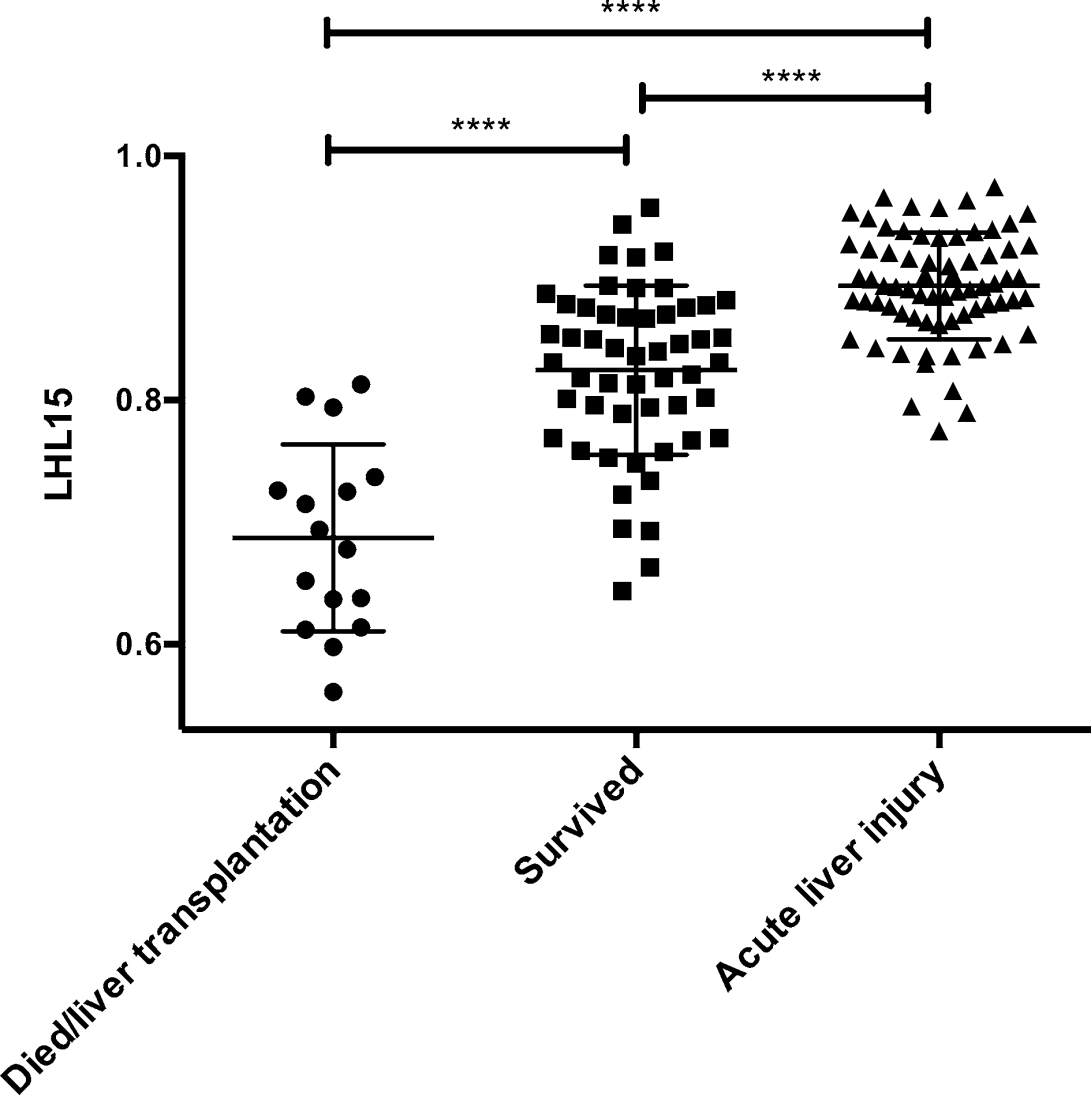
LHL15 levels in patients with severe acute liver injury. Bars indicate medians and standard deviations. For within-group comparisons, a one-way analysis of variance with repeated measures was performed followed by a Bonferroni post-hoc correction. ****P < 0.0001.

Next, the LHL15 values of patients who died or underwent liver transplantation were compared with those of patients who survived (Fig. 2). We also examined Tc-99m GSA scintigraphy in 70 patients with acute liver injury. The mean time for Tc-99m GSA scintigraphy of patients who died or underwent liver transplantation and those who survived was 2.6 (standard deviation, 0.72) and 2.4 (standard deviation, 0.99) days, respectively (*P* = 0.3632). LHL15 values were significantly lower in patients who died or underwent liver transplantation than in those who survived (0.686 vs. 0.836, respectively; *P* < 0.0001). There was a significant negative correlation between the LHL15 and HH15 values (γ =  − 0.8239, *P* < 0.0001; Supplementary Fig. S2). Previously, serial evaluation with Tc-99m GSA scintigraphy was shown to be useful in the evaluation of hepatic function in patients with ALF^23^. Six patients survived despite having an LHL15 of < 0.737 at the time of admission; the LHL15 value of these patients increased significantly during the course of treatment (Supplementary Fig. S3a). Among 16 patients with poor prognosis, 7 underwent chronological re-examination of Tc-99m GSA scintigraphy every 2 weeks after the first examination. The LHL15 values gradually decreased over time, but the changes were not statistically different between the groups (Supplementary Fig. S3b).

### Clinical characteristics of patients who died or underwent liver transplantation and survived

A comparison of the clinical characteristics of patients who died or underwent liver transplantation and those of patients who survived is shown in Table 2. Note that the laboratory tests on admission were analysed. Among 16 patients who died or underwent liver transplantation, 5 developed HE during the clinical course. There were no significant differences in the age, sex, AST, and ALT between those with poor outcomes and those who survived. T-Bil levels, PT-INR, MELD score, and HH15 values were significantly higher, while LHL15 scores were significantly lower in patients who died or underwent liver transplantation than in those who survived. Age, sex, T-Bil levels, PT-INR values, MELD score, and LHL15 values were adopted as confounders for the logistic regression model for multivariate analyses. Multivariate analysis revealed that only LHL15 was an independent predictor of poor outcomes (95% confidence interval [CI], − 0.603 to − 0.016, *P* = 0.0019). A comparison of the day-3 liver test data of patients who died or underwent liver transplantation and those of patients who survived is also shown in Supplementary Table 1. Multivariate analysis using age, sex, day-3 T-Bil levels, day-3 PT-INR values, day-3 MELD score, and LHL15 values revealed that LHL15 (95% CI, − 0.344 to − 0.061, *P* = 0.0088) and day-3 T-Bil levels (95% CI, 0.062 to 0.450, *P* = 0.0229) were independent predictors of poor outcomes.

**Table 2:** Demographic data classified according to outcome.

	Died/liver transplantation (n = 16)	Survived (n = 53)	*P*-value
Age, median (y)	64.5 (50.0–68.8)	56.0 (37.0–67.0)	0.1436
Male:female	9:7	22:31	0.3924
Development of HE during the clinical course	5/16	0/48	0.0006
AST (U/L)	463 (131.8–1318)	1169 (362–2086)	0.1740
ALT (U/L)	637 (94.8–1733.5)	1372 (453–2360.5)	0.0545
Total bilirubin (mg/dL)	18.2 (12.4–21.4)	7.8 (2.7–16.3)	0.0023
PT-INR	2.13 (1.69–3.20)	1.80 (1.58–2.00)	0.0146
MELD score	12.8 (12.4–17.3)	11.0 (8.8–12.2)	< 0.0001
LHL15	0.686 (0.619–0.734)	0.836 (0.779–0.876)	< 0.0001
HH15	0.842 (0.803–0.90)	0.760 (0.702–0.804)	0.0001

Data are presented as *n* (%) or median (interquartile range [IQR]).

*HE* hepatic encephalopathy, *AST* aspartate aminotransferase, *ALT* alanine aminotransferase, *PT-INR* prothrombin time-international normalized ratio, *MELD* model for end-stage liver disease, *LHL15* Hepatic accumulation index calculated by dividing the radioactivity of the liver regions of interest by that of the liver-plus-heart regions of interest at 15 min. HH15, Blood clearance index calculated by dividing the radioactivity of the heart regions of interest at 15 min by that at 3 min.

### Comparison of the 6-month transplant-free survival rates between the two groups stratified based on the LHL15 cut-off value for the prediction of prognosis

**Figure 3:**
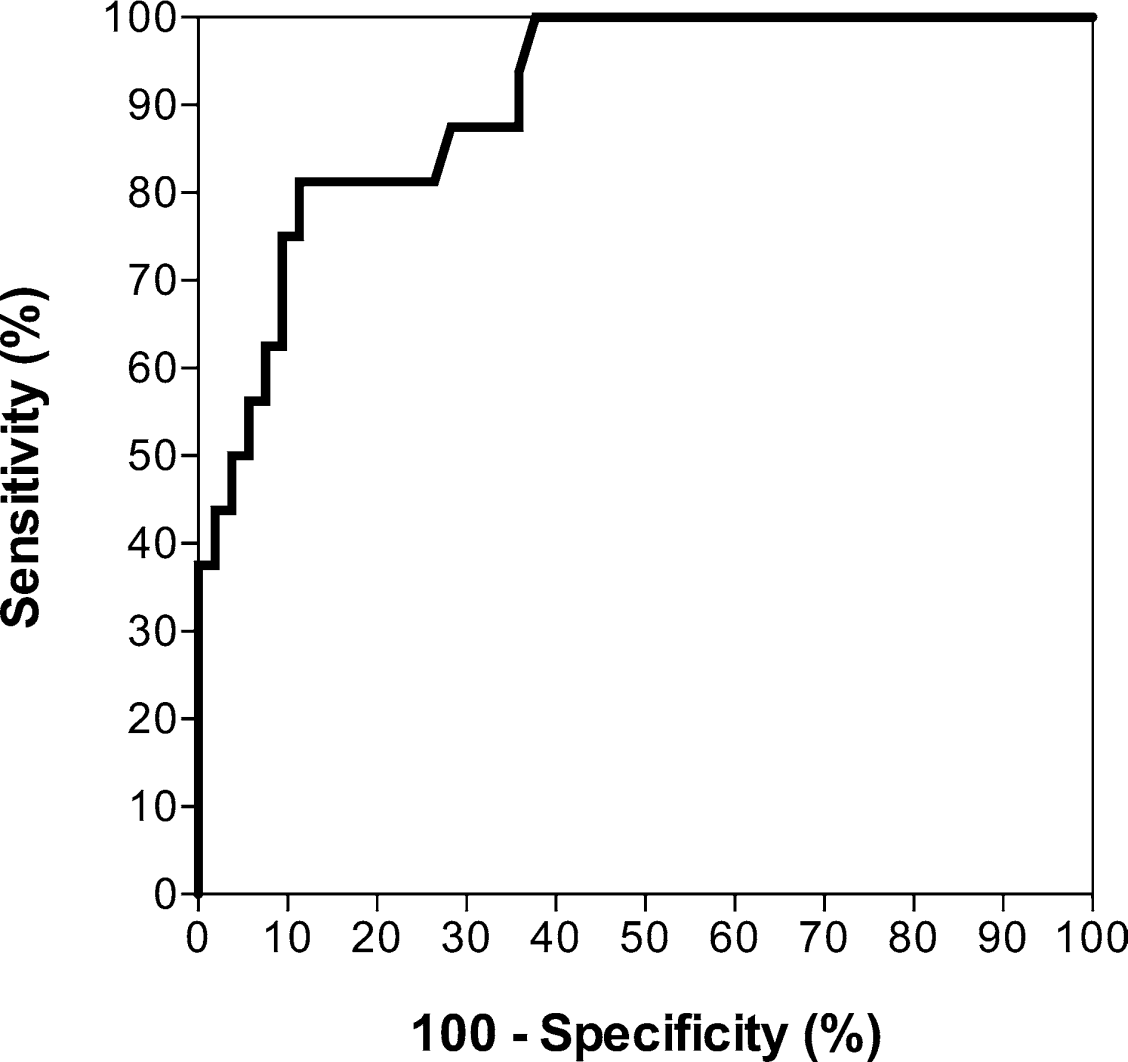
Receiver operating characteristic curve for predicting death or requiring liver transplantation via LHL15 from Tc-99m GSA scintigraphy obtained within 3 days after admission.

**Figure 4:**
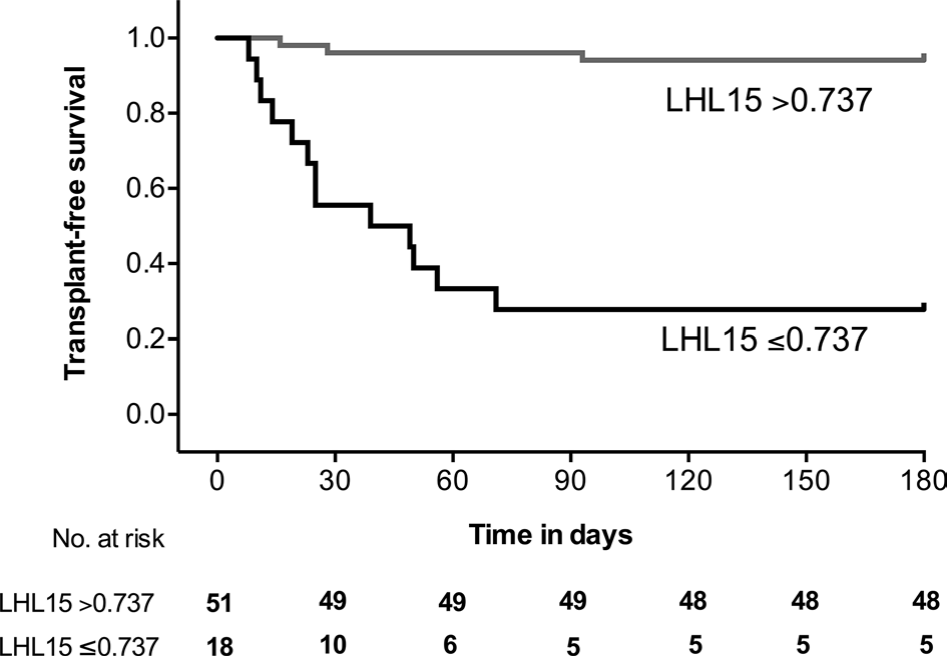
Survival curves stratified according to LHL15 levels according to the optimal cut-off values in patients with severe acute liver injury.

The optimal cut-off value for LHL15 to predict the prognosis of patients who died or underwent liver transplantation and those who survived was calculated using Youden’s index as 0.737 with a sensitivity of 81.3%, specificity of 88.7%, and area under the curve (AUC) of 0.907 (95% CI, 0.832–0.981; Fig. 3). The AUC of MELD score, PT-INR, T-Bil, and ALT with regard to predicting the prognosis were 0.849 (95% CI, 0.740–0.919), 0.669 (95% CI, 0.489–0.810), 0.764 (95% CI, 0.610–0.871), and 0.688 (95% CI, 0.518–0.820), respectively (Supplementary Fig. S4). The AUC of the LHL15 was significantly higher than the PT-INR and ALT (*P* = 0.0040, *P* = 0.0126, respectively). There was no statistically significant difference in the AUC of the LHL15 between the MELD score and PT-INR (*P* = 0.0509, *P* = 0.2990, respectively). At the LHL15 cut-off, the positive and negative predictive values were 68.4% and 94.0%, respectively. Based on the LHL15 cut-off value of 0.737, the 6-month transplant-free survival rates of patients with an LHL15 level of > 0.737 were compared with those of patients with an LHL15 level of ≤ 0.737 (Fig. 4). The 6-month transplant-free survival rate was 94.0% in patients with an LHL15 level of > 0.737, whereas the transplant-free survival rate was 31.6% in patients with an LHL15 level of ≤ 0.737. The 6-month transplant-free survival rate of patients with an LHL15 level ≤ 0.737 was significantly lower than that of patients with an LHL15 level of > 0.737 (log-rank test, *P* < 0.0001; hazard ratio 19.07; 95% CI, 19.55–243.70).

## Discussion

The present study demonstrated that Tc-99m GSA scintigraphy can help identify SLI patients who are at risk of death or require liver transplantation. Tc-99m GSA scintigraphy could be a reliable method for predicting outcomes in patients with fulminant hepatic failure^16^ as well as those with cirrhosis^17^. However, its efficacy in predicting the outcomes of patients with SLI has not yet been examined. Herein, the LHL15 values were significantly lower in patients who died or underwent liver transplantation than in those who survived. Based on the results of the comparison of 6-month transplant-free survival rates between patients with an LHL15 level of > 0.737 and those with an LHL15 level of ≤ 0.737, the risk of death or requiring liver transplantation was more than 19-fold higher among patients with an LHL15 level of ≤ 0.737 compared to those with an LHL15 level of > 0.737. The majority of patients with an LHL15 level of ≤ 0.737 died or required liver transplantation (12 out of 13) within 60 days after admission, suggesting that the evaluation of liver transplantation as a therapeutic option was likely considered for these patients.

SLI patients usually recover spontaneously without any specific treatment because of the remarkable regenerative capacity of the liver. However, some SLI patients progress to ALF with varying degrees of HE^1,12^. ALF is associated with high mortality and can be substantially reduced by liver transplantation. Therefore, distinguishing SLI patients who might progress to ALF and are at risk of death is important when evaluating patients requiring liver transplantation. A previous prospective study of 68 consecutive patients with mixed etiologies and SLI (defined as a PT-INR > 1.7) found that 12% of patients died or underwent liver transplantation^10^. In our previous prospective study, 29.3% of SLI patients progressed to ALF, followed by death or liver transplantation^12^. It is universally acknowledged that the transfer of SLI patients to a transplant center after the onset of HE is associated with a deteriorated outcome^24^. To prevent these circumstances, we developed a model to predict HE in acute hepatitis patients, which is to be followed by permission to transfer patients with a poor prognosis to specialized centers for liver transplantation^12^. The present study demonstrates that Tc-99m GSA scintigraphy data are useful for the early identification of SLI patients with poor outcomes. Although Tc-99m GSA scintigraphy cannot be performed at every facility, it is a promising tool for the assessment of liver function at specialized centers.

To assess the severity of liver injury and hepatic function, the PT-INR was monitored in SLI patients, as in patients with other liver diseases. In SLI patients, coagulopathy is treated with fresh frozen plasma in the event of bleeding or a planned invasive procedure^25,26^. Correction of coagulopathy by administration of fresh frozen plasma alters the PT, which interferes with the assessment of coagulation status. Because Tc-99m GSA scintigraphy directly reflects the function of hepatocytes, it provides a reliable evaluation of hepatic function without being affected by the use of fresh frozen plasma.

The present study had several limitations. First, no SLI caused by acetaminophen overdose was reported during the observational period; therefore, Tc-99m GSA scintigraphy data for acetaminophen-induced SLI was not evaluated. In the United States and Western Europe, acetaminophen overdose is the predominant cause of acute liver injury, SLI, and ALF^1,27,28^. SLI caused by acetaminophen overdose has a more favorable outcome than SLI with non-acetaminophen etiologies^1,24^. Further studies are needed to evaluate whether Tc-99m GSA scintigraphy can also predict the prognosis of acetaminophen-induced liver injury. Second, LHL15 values calculated using Tc-99m GSA scintigraphy were influenced by liver function at the time of admission. In other words, the LHL15 value might be higher in patients who have been transported to the hospital prior to the deterioration of liver function. Likewise, if a patient is transported after liver function has deteriorated, the LHL15 value might be lower. In the current study, LHL15 values decreased gradually in patients who died or underwent liver transplantation (Supplementary Fig. S3b), suggesting that the longitudinal evaluation of Tc-99m GSA scintigraphy is also beneficial for monitoring hepatic function. The optimal interval for conducting re-inspections is a subject for future studies. Third, patients in critical conditions, especially ALF patients with severe HE or requiring mechanical ventilation, cannot be transferred to the Tc-99m GSA scintigraphy inspection room. However, patients who progress to a severe state beforehand do not require Tc-99m GSA scintigraphy because their prognosis is poor^29^. Tc-99m GSA scintigraphy is useful for predicting whether hepatic function will deteriorate when the present condition is relatively good.

In conclusion, the present study demonstrated that Tc-99m GSA scintigraphy obtained within the first 3 days after hospital admission may provide early objective information for predicting the prognosis of patients with SLI. In clinical practice, Tc-99m GSA scintigraphy may be an adjunctive diagnostic tool for indicating SLI patients at risk of progressing to ALF and requiring liver transplantation.

## Supplementary Information

Supplementary Information 1.

Supplementary Information 2.

## Abbreviations

Tc-99m GSA: Technetium-99m-diethylenetriaminepentaacetic acid galactosyl human serum albumin
SLI: Severe acute liver injury
ALF: Acute liver failure
HE: Hepatic encephalopathy
AST: Aspartate aminotransferase
ALT: Alanine aminotransferase
PT-INR: Prothrombin time-international normalized ratio
ROI: Regions of interest
MELD: Model for end-stage liver disease
Cre: Creatinine
T-Bil: Total bilirubin
HBV: Hepatitis B virus
CI: Confidence interval
AUC: Area under the curve
