# Supplementary material for: Tc-99m GSA scintigraphy within the first 3 days after admission as an early predictor of outcome in severe acute liver injury
Source: Sci Rep. 2021 Jun 15;11:12518. doi: 10.1038/s41598-021-92058-6 (PMC8206118; doi:10.1038/s41598-021-92058-6)
Supplement: Supplementary file 1 — Supplementary Information 1. [file 41598_2021_92058_MOESM1_ESM.pdf]

Tc-99m GSA scintigraphy within the first 3 d after admission as an early predictor of outcome in severe acute liver injury

Yuji Suzuki MD, PhD*; Keisuke Kakisaka MD, PhD; Takuro Sato MD, PhD; Ryouichi Mikami MD; Hiroaki Abe MD; Tokio Sasaki MD, PhD; and Yasuhiro Takikawa MD, PhD*

Division of Hepatology, Department of Internal Medicine, Iwate Medical University School of Medicine, Yahaba, Iwate, Japan

Correspondence to:

Yuji Suzuki, Yasuhiro Takikawa

Division of Hepatology, Department of Internal Medicine, Iwate Medical University School of Medicine, 1-1-1 Idaidori, Yahaba-cho, Shiwa-gun, Iwate 028-3694, Japan

Tel: +81-19-651-5111

Fax: +81-19-907-7166

E-mail: yusuzuki@iwate-med.ac.jp, ytakikaw@iwate-med.ac.jp

Supplementary Figures

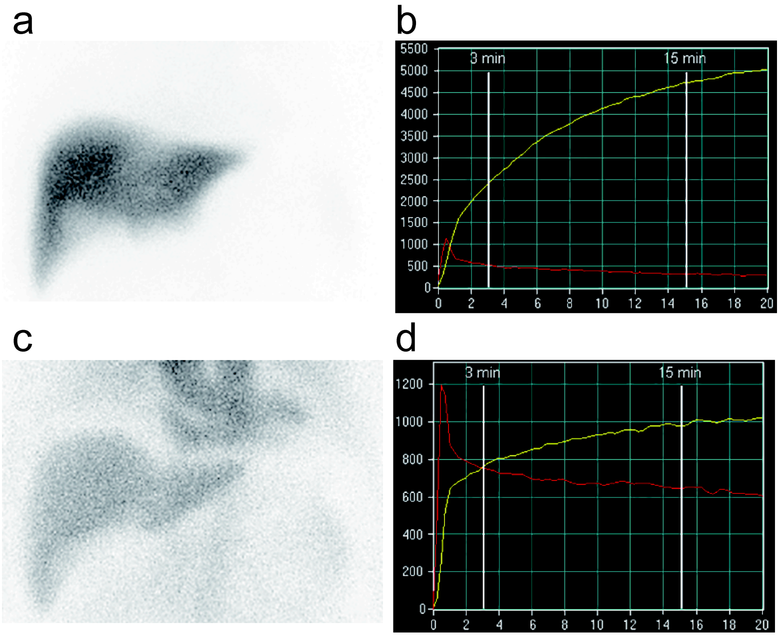

Supplementary Figure 1. (**a**) Tc-99m GSA scintigraphy in a patient with a normal LHL15 value. Anterior planar image 15 min after injection of Tc-99m GSA showing radioisotope accumulation in the liver. (**b**) Tc-99m GSA time-activity curves from the heart (red) and liver (yellow). The LHL15 was calculated as the liver counts at 15 min divided by the heart counts-plus-liver counts at 15 min. Additionally, the HH15 was calculated as the heart count at 15 min divided by the heart counts at 3 min. (**c**) Typical Tc-99m GSA scintigraphy anterior planar image 15 min after injection in a patient with severe acute liver injury. (**d**) Tc-99m GSA time-activity curves from the heart (red) and liver (yellow). The radioisotope uptake into the liver is impaired, and stagnation of the radioisotope in the heart has occurred.

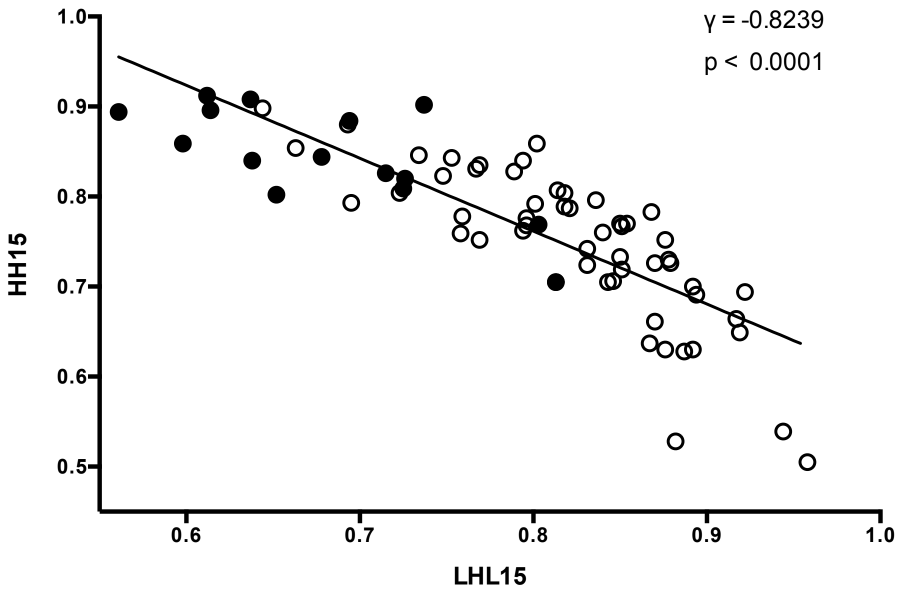

Supplementary Figure 2. Correlation between LHL15 and HH15 values in SLI patients ● = Patients who died or underwent liver transplantation

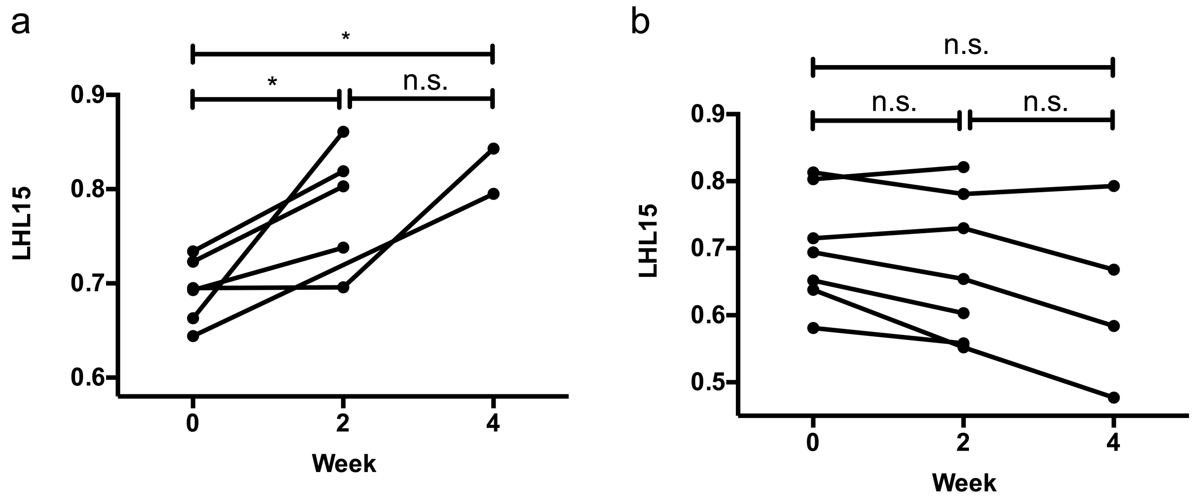

Supplementary Figure 3. Dynamic changes in LHL15 values during the treatment course for severe acute liver injury. (**a**) Serial changes in LHL15 values in patients who survived despite having LHL15 values < 0.737 at the time of admission. (**b**) Serial changes in LHL15 values that were re-examined in patients with poor prognosis. For within-group comparisons, a one-way analysis of variance with repeated measures was performed followed by a Bonferroni post-hoc correction. *$P < 0.05$, n.s., not significant

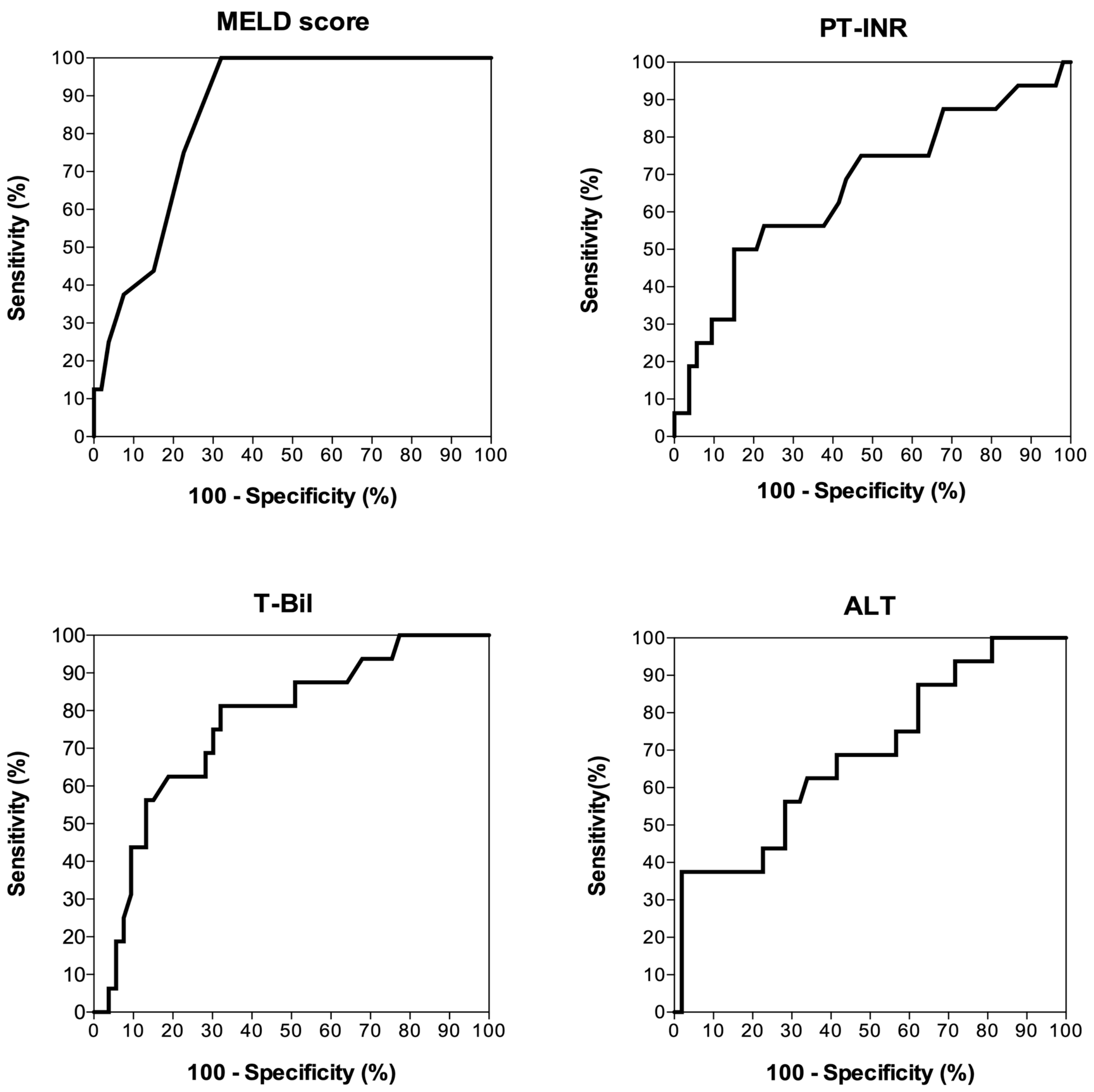

Supplementary Figure 4. Area under the curve analysis for prediction of prognosis for MELD score, PT-INR, T-Bil, and ALT. MELD, Model for End-Stage Liver Disease; PT-INR, prothrombin time-international normalized ratio; T-Bil, total bilirubin; ALT, alanine aminotransferase.
