# Supplementary material for: Tc-99m GSA scintigraphy within the first 3 days after admission as an early predictor of outcome in severe acute liver injury
Source: Sci Rep. 2021 Jun 15;11:12518. doi: 10.1038/s41598-021-92058-6 (PMC8206118; doi:10.1038/s41598-021-92058-6)
Supplement: Supplementary file 2 — Supplementary Information 2. [file 41598_2021_92058_MOESM2_ESM.pdf]

Supplementary Table 1. The third day liver tests data classified according to outcome

	Died/liver transplantation (n = 16)	Survived (n = 53)	*P*-value
AST (U/L)	150 (66.3–428)	250 (144–702.5)	0.3573
ALT (U/L)	356 (92.8–1229)	715 (307.5–1656)	0.4135
Total bilirubin (mg/dL)	16.1 (12.2–20.4)	6.8 (3.1–13.7)	0.0074
PT-INR	1.95 (1.69–3.08)	1.56 (1.33–1.73)	0.0254
MELD score	12.5 (11.2–14.0)	10.8 (7.8–12.7)	0.0103

AST, aspartate aminotransferase; ALT, alanine aminotransferase; PT-INR, prothrombin time-international normalized ratio; MELD, Model for End-stage Liver Disease. Data are presented as median (interquartile range [IQR]).
